# 13-Ethylberberine Induces Apoptosis through the Mitochondria-Related Apoptotic Pathway in Radiotherapy-Resistant Breast Cancer Cells

**DOI:** 10.3390/molecules24132448

**Published:** 2019-07-04

**Authors:** Hana Jin, Young Shin Ko, Sang Won Park, Ki Churl Chang, Hye Jung Kim

**Affiliations:** Department of Pharmacology, College of Medicine, Institute of Health Sciences, Gyeongsang National University, Jinju 52727, Korea

**Keywords:** apoptosis, 13-ethylberberine, mitochondrial ROS, RT-R breast cancer cells

## Abstract

Berberine is reported to have multiple biological effects, including antimicrobial, anti-inflammatory, and antitumor activities, and 13-alkyl-substituted berberines show higher activity than berberine against certain bacterial species and human cancer cell lines. In particular, 13-ethylberberine (13-EBR) was reported to have anti-inflammatory effects in endotoxin-activated macrophage and septic mouse models. Thus, in this study, we aimed to examine the anticancer effects of 13-EBR and its mechanisms in radiotherapy-resistant (RT-R) MDA-MB-231 cells derived from the highly metastatic MDA-MB-231 cells. When we compared the gene expression between MDA-MB-231 and RT-R MDA-MB-231 cells with an RNA microarray, RT-R MDA-MB-231 showed higher levels of anti-apoptotic genes and lower levels of pro-apoptotic genes compared to MDA-MB-231 cells. Accordingly, we examined the effect of 13-EBR on the induction of apoptosis in RT-R MDA-MB-231 and MDA-MB-231 cells. The results showed that 13-EBR reduced the proliferation and colony-forming ability of both MDA-MB-231 and RT-R MDA-MB-231 cells. Moreover, 13-EBR induced apoptosis by promoting both intracellular and mitochondrial reactive oxygen species (ROS) and by regulating the apoptosis-related proteins involved in the intrinsic pathway, not in the extrinsic pathway. These results suggest that 13-EBR has pro-apoptotic effects in RT-R MDA-MB-231 and MDA-MB-231 cells by inducing mitochondrial ROS production and activating the mitochondrial apoptotic pathway, providing useful insights into new potential therapeutic strategies for RT-R breast cancer treatment.

## 1. Introduction

Breast cancer is one of the most common causes of death in women around the world [1]. Most breast cancer patients respond to conventional treatments such as surgery, chemotherapy, and radiotherapy. However, there are inherent limitations to each therapy, which cause therapeutic resistance and the relapse of cancer, eventually leading to the failure of therapy. In particular, radiotherapy has several benefits, such as improving overall survival and synergizing with surgical resection [2,3]; the relapse of cancer after radiotherapy is common and, in particular, ductal carcinoma and early/advanced invasive breast cancers show radiotherapy resistance. Moreover, because triple-negative breast cancer (TNBC), which is characterized by the absence of estrogen receptor (ER), progesterone receptor (PR), and human epidermal growth factor receptor 2 (HER2) expression, is an aggressive type of cancer that is difficult to treat, breast cancer patients suffer more if TNBC acquires radiotherapy resistance, and the patients do not survive after radiation therapy. Therefore, in a previous study, we repeatedly irradiated the MDA-MB-231 breast cancer cell line, which is a common cell model system to represent highly metastatic TNBC, to establish radiotherapy-resistant MDA-MB-231 (RT-R MDA-MB-231) cells, and we aimed to find effective therapeutics to treat RT-R MDA-MB-231 cells in the present study.

Berberine (BBR) is an isoquinoline alkaloid that is isolated from *Cotridis rhizoma* and has multiple biological activities, such as antimicrobial, anti-inflammatory, and antitumor effects [4,5,6,7]. In particular, the anticancer effects of BBR on breast cancer cells were reported; BBR induces breast cancer cell apoptosis via the activation of the apoptotic signaling pathway [8,9], the inhibition of proliferation and migration [10], the suppression of cell motility through the downregulation of related molecules [11,12], and the enhancement of chemosensitivity, which induces apoptosis [13]. Recently, it was reported that 13-alkyl-substituted berberines showed better antimicrobial activity against certain bacterial species and cytotoxic activity against human cancer cell lines than BBR [14,15]. Furthermore, among these 13-alkyl-substituted berberines, 13-ethylberberine (13-EBR) was reported to have anti-inflammatory effects in endotoxin-activated macrophage and septic mouse models [16,17]. However, the effects of 13-EBR on cancer cell growth and signaling pathways were not reported. Therefore, we tried to identify the differences between MDA-MB-231 cells and RT-R MDA-MB-231 cells in gene expression levels, and determined the anticancer effects of 13-EBR on RT-R MDA-MB-231 breast cancer cells, as well as MDA-MB-231. Moreover, we explored the associated mechanisms of 13-EBR using MDA-MB-231 and RT-R MDA-MB-231 breast cancer cells in this study.

## 2. Results

### 2.1. 13-EBR Had Anticancer Effects on RT-R MDA-MB-231 Cells and MDA-MB-231 Cells, as Demonstrated by Suppressing the Proliferation and Colony-Forming Ability

**Figure 1:**
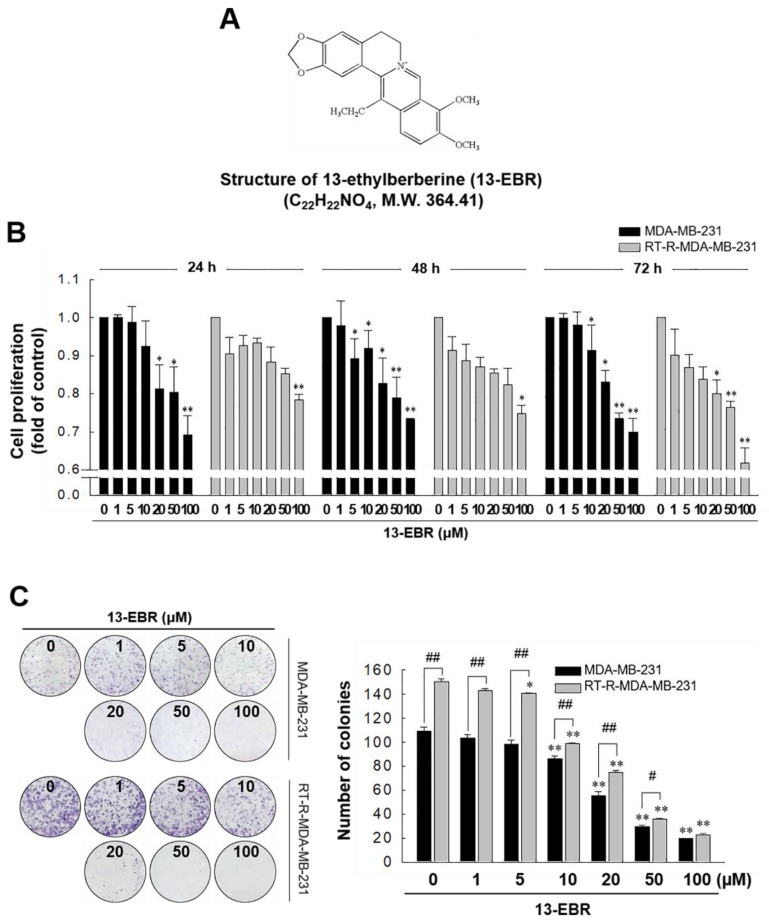
Chemical structure of 13-ethylberberine (13-EBR), and the effects of 13-EBR on cell proliferation, colony formation, and apoptosis in breast cancer cells. (**A**) The chemical structure of 13-EBR. (**B**) MDA-MB 231 and radiotherapy-resistant (RT-R) MDA-MB 231 cells were treated with 13-EBR at the indicated doses (1, 5, 10, 20, 50, and 100 µM) for 24–72 h, and cell proliferation was measured using the Cell Counting Kit-8 (CCK-8) reagent, as described in Section 4. The values represent the mean ± standard error of the mean (SEM) of three independent experiments; ^**^ *p* < 0.01, ^*^ *p* < 0.05 compared to the controls (vehicle-treated cells) at each time point. (**C**) Both breast cancer cell lines (1000 cells/well) were seeded in six-well plates. The cells were stimulated with 13-EBR for 24 h at the indicated doses. Following treatment, a colony-formation assay was performed, as described in Section 4, and was quantified by counting the colonies. The values represent the mean ± SEM of three independent experiments; ^**^ *p* < 0.01, ^*^ *p* < 0.05 compared to the control for each cell line; ^##^ *p* < 0.01, ^#^ *p* < 0.05 compared to the MDA-MB-231 cells.

In our previous study, we showed that RT-R MDA-MB-231 cells had increased cell viability and colony-forming ability after irradiation, and exhibited higher chemoresistance compared to the MDA-MB-231 parental cells [18]. In this study, we analyzed the gene expression levels between MDA-MB-231 cells and RT-R MDA-MB-231 cells and found that RT-R MDA-MB-231 cells showed lower expression of pro-apoptotic genes and higher expression of anti-apoptotic genes than MDA-MB-231 cells (Table 1). Thus, we were interested in identifying effective anticancer drugs to treat RT-R breast cancer cells because numerous cancer patients suffer from aggressive disease and the relapse of radiotherapy-resistant cancer. Figure 1 shows that 13-EBR effectively reduced proliferation (Figure 1B) and colony formation (Figure 1C) in RT-R MDA-MB-231 cells and MDA-MB-231 cells in a dose-dependent manner compared to the controls. These results suggested that 13-EBR has anticancer effects as a result of the suppression of cell growth and colony-forming ability in both MDA-MB-231 and RT-R MDA-MB-231 cells.

### 2.2. 13-EBR Upregulated Intracellular Total and Mitochondrial ROS Production in Both MDA-MB-231 and RT-R MDA-MB-231 Cells

**Figure 2:**
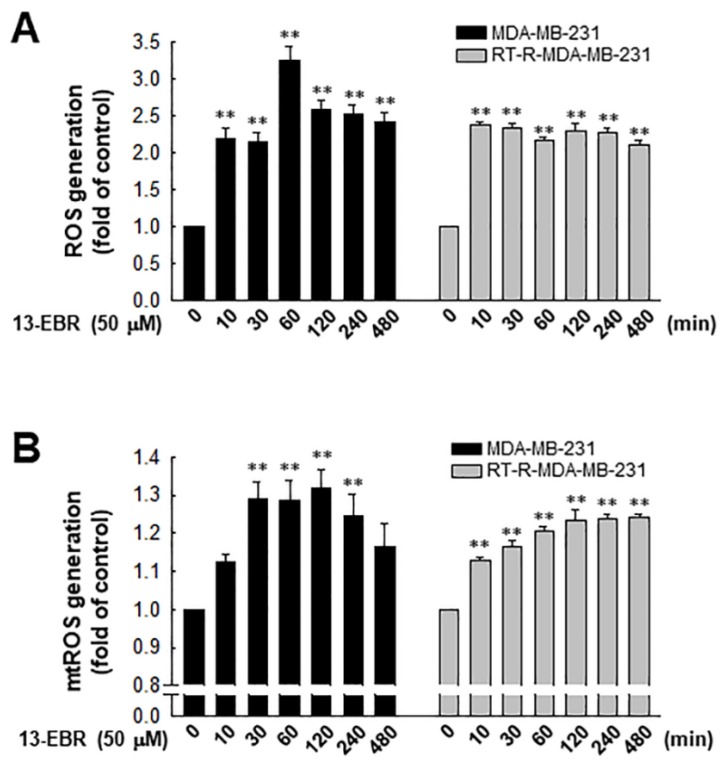
Effects of 13-EBR on the production of intracellular and mitochondrial reactive oxygen species (ROS) production in MDA-MB-231 and RT-R MDA-MB-231 cells. (**A**,**B**) Both cell lines were stimulated with 50 µM 13-EBR for the indicated times, and then the intracellular ROS (**A**) and mitochondrial ROS (**B**) were measured by staining with 2’,7’-dichlorodihydrofluorescein diacetate (H_2_DCF-DA) and MitoSOX Red, respectively. The values represent the mean ± SEM of three independent experiments; ^**^ *p* < 0.01 compared to the control of each cell line.

It was reported that excessive ROS can induce apoptosis through both the extrinsic and intrinsic pathways [19]. Furthermore, excessive mitochondrial oxidant stress can induce cell death in tumor cells [20]. Thus, we examined the effects of 13-EBR on the production of intracellular total ROS, including mitochondrial ROS, in RT-R MDA-MB-231 and MDA-MB-231 cells. When both of the breast cancer cell lines were treated with 50 µM 13-EBR, the treatment significantly enhanced the intracellular total ROS and mitochondrial ROS production from early time points compared to the controls (Figure 2).

### 2.3. 13-EBR Induced MDA-MB-231 and RT-R MDA-MB-231 Apoptosis through a Mitochondria-Related Apoptotic Pathway, Not an Extrinsic Pathway

**Figure 3:**
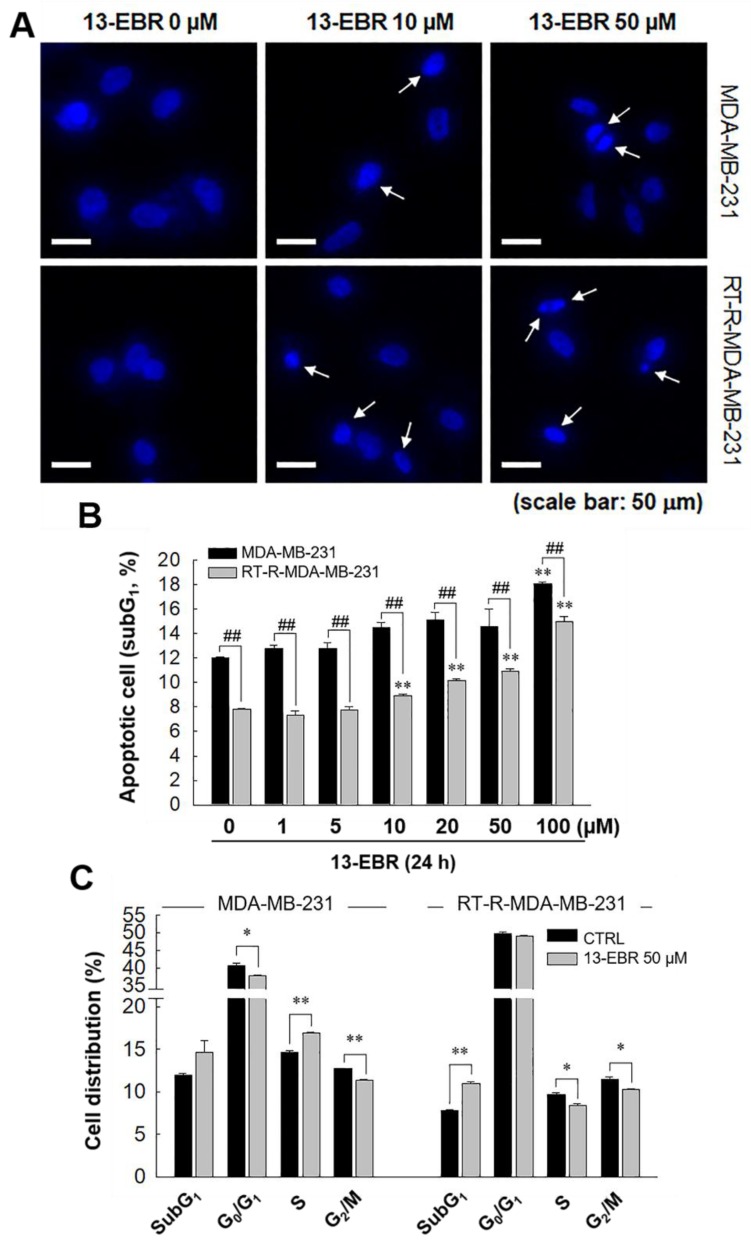
Induction of apoptotic cell death in MDA-MB-231 and RT-R MDA-MB-231 cells with 13-EBR through the intrinsic pathway. (**A**) Both cell lines were seeded on a cover slip that was mounted onto a self-designed perfusion chamber and then stimulated with 13-EBR at the indicated doses for 24 h. The fragmented DNA was observed, as described in Section 4. White arrows represent the fragmented DNA. (**B**) Both breast cancer cell lines were treated with 13-EBR at the indicated doses for 24 h, and then apoptotic cells were identified by analyzing the sub-gap 1 (subG_1_) phase using a fluorescence-activated cell sorting (FACS) system, as described in Section 4. (**C**) Both cell lines were treated or were not treated with 13-EBR at 50 µM for 24 h, and then the cell distribution in the cell cycle was determined using the FACS system. The values represent the mean ± SEM of three independent experiments; ^**^ *p* < 0.01, ^*^ *p* < 0.05 compared to the control; ^##^ *P* < 0.01 compared to the MDA-MB-231 cells.

**Figure 4:**
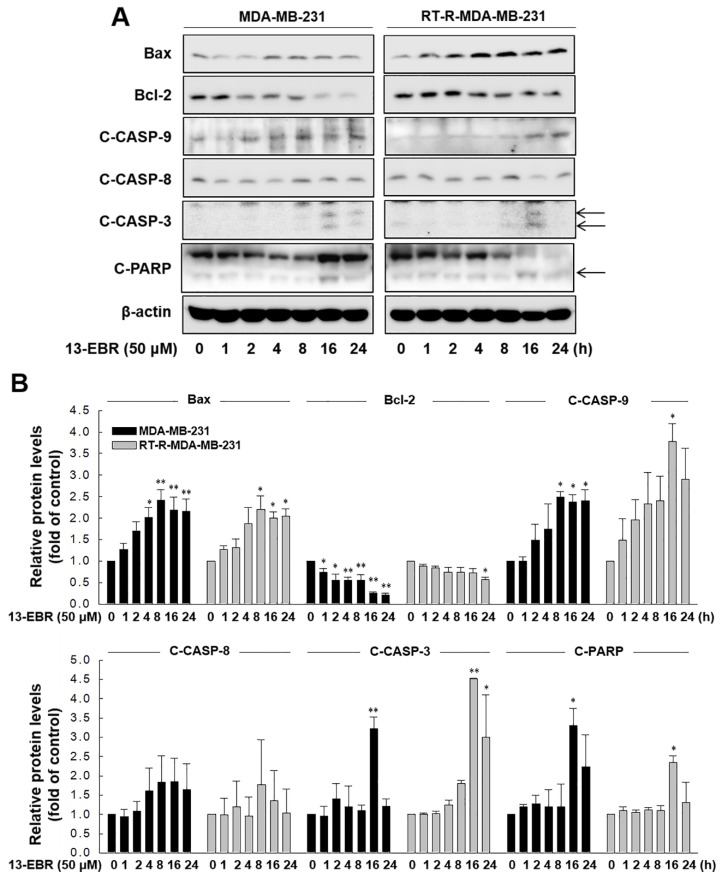
13-EBR-mediated induction of apoptosis through the intrinsic pathway. (**A**,**B**) Both cell lines were stimulated with 50 µM 13-EBR for the indicated times, and total proteins were extracted. The Bax, Bcl-2, cleaved caspase-9 (C-CASP-9), -8 (C-CASP-8), -3 (C-CASP-3), cleaved poly(ADP ribose) polymerase (C-PARP), and β-actin protein levels were analyzed in cell lysates by Western blotting (**A**), as described in Section 4, and were quantified (**B**). The values represent the mean ± SEM of three independent experiments; ^**^ *p* < 0.01, ^*^ *p* < 0.05 compared to the control of each cell line.

Next, we further examined whether 13-EBR induces apoptosis in both MDA-MB-231 and RT-R MDA-MB-231 cells by observing DNA shrinkage or nuclear fragmentation that occurs in cells undergoing apoptosis. As expected, 13-EBR stimulation induced DNA shrinkage at 10 μM and DNA fragmentation at 50 μM in both MDA-MB-232 and RT-R MDA-MB-231 cells (Figure 3A). Moreover, 13-EBR induced apoptosis, as shown by the increased sub-gap 1 (subG_1_) population in both MDA-MB-231 and RT-R MDA-MB-231 cells in a dose-dependent manner compared to that in the controls (Figure 3B). In addition to the subG_1_ population, the synthesis (S) and gap 2/mitosis (G_2_/M) populations were remarkably changed in response to 50 μM 13-EBR treatment in MDA-MB-231 and RT-R MDA-MB-231 cells compared to the controls (Figure 3C). As mentioned above, an increase in ROS can induce apoptosis through both the extrinsic and intrinsic pathways. Thus, we investigated which apoptotic signaling pathway is involved in 13-EBR-induced apoptosis in MDA-MB-231 and RT-R MDA-MB-231 cells. The protein level of Bax, a pro-apoptotic gene, in the control group of RT-R MDA-MB-231 was little lower than that in the control of MDA-MB-231, as presented in Table 1, and 13-EBR stimulation significantly increased the protein level of Bax in both cell lines. However, 13-EBR decreased the level of *Bcl-2*, an anti-apoptotic gene, in a time-dependent manner compared to that in the controls in both cell lines. Moreover, the protein levels of cleaved caspase-9, -3, and poly(ADP ribose) polymerase (PARP) were also increased in response to 13-EBR treatment compared to the controls. However, 13-EBR did not affect the cleaved caspase-8 protein levels (Figure 4A,B). These results suggested that 13-EBR induces apoptotic cell death via the regulation of the mitochondria-related intrinsic pathway rather than an extrinsic pathway in MDA-MB-231 and RT-R MDA-MB-231 cells.

## 3. Discussion

TNBC refers to breast cancer that does not express ER, PR, and HER2, which is known to be more aggressive, with worse prognosis than that of other types of breast cancers that express hormone receptors [21,22]. Due to the lack of specific molecular targets, general cancer treatments are limited and not available for the treatment of TNBC patients. Thus, combinatorial therapy, which consists of surgery, chemotherapy, and radiation, is used for TNBC patients. According to clinically safe and effective therapeutic methods, a small amount of X-ray is periodically used to irradiate breast cancer, and even with combinatorial treatment, the surviving residual cancer cells eventually exhibit radiotherapy resistance [23]. Previously, we reported that not only the RT-R TNBC cell line (RT-R MDA-MB-231) but also the ER- and PR-positive breast cancer cell lines (RT-R MCF-7 and RT-R T47D) showed increased proliferation and colony formation, and were even more resistant to cancer chemotherapy than their parental breast cancer cells. Among these cells, RT-R MDA-MB-231 cells exhibited notable aggressiveness during tumor growth and invasion and showed characteristics of breast cancer stem cells [18]. Therefore, we tried to find a possible anticancer drug candidate, and, in this study, we investigated the anticancer effect of 13-EBR and the underlying mechanisms in radiotherapy-resistant TNBC.

BBR is known to be a low-toxicity and safe agent and was reported to have multiple biological functions, including antimicrobial, anti-inflammatory, and antitumor effects [4,5,6,7,8,9,10,11,12,13]. Treatment with 13-EBR, a BBR analog that is substituted at C-13 by alkyl groups, was reported to have anti-inflammatory effects [16,17]; however, the role of this molecule in tumor suppression was not reported. Thus, we aimed to determine whether 13-EBR could be a potential chemotherapeutic agent by examining the effects of 13-EBR on MDA-MB-231 cells and the radiotherapy-resistant TNBC cell line, RT-R MDA-MB-231. In this study, our results revealed that 13-EBR suppressed RT-R MDA-MB-231 and MDA-MB-231 cell proliferation and colony-forming ability (Figure 1) compared to the controls. Further studies showed that 13-EBR stimulation induced the production of ROS, including mitochondrial ROS, in both breast cancer cell lines (Figure 2). In addition, 13-EBR caused cell-cycle arrest and upregulated Bcl-2 family protein levels, such as Bax (pro-apoptotic), and reduced Bcl-2 (anti-apoptotic) levels and activated the caspase-9 and -3 pathways but not the caspase-8 pathway, suggesting that 13-EBR evokes apoptosis through the mitochondria-mediated signaling pathway in RT-R MDA-MB-231 cells (Figure 3). Although it was reported that 13-alkyl-substituted berberines showed better anti-microbe and anticancer activities than BBR [14,15], as described in Section 1, there is no evidence about which one of the two compounds has better efficacy on suppressing breast cancer cells, especially RT-R TNBC. Thus, we further compared the effects of BBR and 13-EBR on proliferation, colony formation, and cellular apoptosis in RT-R MDA-MB-231 cells. Interestingly, 13-EBR was significantly more effective in suppressing the cell proliferation and colony-forming ability and in inducing cellular apoptosis than BBR in RT-R MDA-MB-231 cells, which showed a significantly enhanced ability of colony formation and lower levels of apoptotic cell population compared to MDA-MB-231 cells (Supplementary Materials). When we determined the toxicity of 13-EBR on normal epithelial cells (MCF-10A), 13-EBR showed cytotoxicity in a dose-dependent manner; however, 20 μM 13-EBR, which showed apoptotic cell death and anti-colony forming ability in MDA-MB-231 and RT-R MDA-MB-231, showed less toxicity (cell viability over than 80%) (data not shown).

Many chemotherapeutic and radiotherapeutic agents eliminate cancer cells through the augmentation of ROS production [24,25]. A high level of mitochondrial ROS can also initiate intrinsic apoptosis, leading to the release of cytochrome c, a mitochondrial apoptogenic factor, into the cytosol [26]. In this study, we determined that 13-EBR increases the level of intracellular total ROS, including mitochondrial ROS, compared to that in the controls (Figure 2). Furthermore, cell-cycle progression is linked to cell proliferation and apoptosis. The cell cycle is divided into four phases, and the cellular decision to initiate mitosis or to be quiescent (G_0_ state) occurs during the G_1_ phase [27]. Thus, we determined whether 13-EBR acts as a regulator of the cell cycle in breast cancer cells, and elicited a cell-cycle arrest in the subG_1_ phase, which was increased in response to 13-EBR (Figure 3B,C) compared to that in the controls, suggesting that 13-EBR functions as an apoptosis inducer in RT-R TNBC and TNBC.

Apoptosis is essential for normal development and tissue homeostasis, and perturbations in the regulation of apoptosis contribute to numerous pathological conditions, including cancer, autoimmune diseases, and degenerative diseases [28]. Furthermore, apoptosis is an important target for anticancer drugs because accumulated evidence on cancer development indicated that cancerous cells are able to survive due to acquired mechanisms of apoptosis resistance in addition to uncontrolled proliferation [28]. Apoptosis is a form of programmed cell death that can be initiated through one of two of the best-understood activation mechanisms: extrinsic and intrinsic pathways [29]. The extrinsic signaling pathways that initiate apoptosis involve transmembrane receptor-mediated interactions. The extrinsic pathway-related death receptors are members of the tumor necrosis factor (TNF) receptor gene superfamily, such as cluster of differentiation 95 (CD95) (apoptosis antigen-1; APO-1/Fas) or TNF-related apoptosis-inducing ligand (TRAIL) receptors [30]. Upon ligand binding, the death receptors trigger the activation of the initiator caspase-8, and, once caspase-8 is activated, the execution phase of apoptosis is triggered through the direct cleavage of downstream effector caspases, such as caspase-3 [30]. On the other hand, the intrinsic signaling pathways that initiate apoptosis related to diverse nonreceptor-mediated stimuli produce intracellular signals that act directly on targets within the cell, which are mitochondrial-initiated events. The stress stimuli that can trigger the intrinsic pathway can occur in the absence of certain growth factors, cytokines, toxins, hypoxia, hyperthermia, viral infections, and free radicals [31]. The members of the Bcl-2 family of proteins mediate these apoptotic mitochondrial events. The Bcl-2 family proteins control mitochondrial membrane permeability and can be either pro-apoptotic or anti-apoptotic. Some of the anti-apoptotic proteins include Bcl-2, Bcl-XL, Bcl-XS, and Bcl-2 associated athanogene (BAG); some of the pro-apoptotic proteins include Bax, Bak, Bid, Bad, and Bim. The main mechanism of action of Bcl-2 family proteins is the regulation of cytochrome c release from the mitochondria via the alteration of mitochondrial membrane permeability [32]. Because stimulation with 13-EBR caused apoptotic cell death in MDA-MB-231 and RT-R MDA-MB-231 cells, we examined which apoptotic signaling pathway is involved in the 13-EBR-mediated induction of apoptosis and tried to investigate how 13-EBR affects RT-R MDA-MB-231 cells, which had lower expression of pro-apoptotic genes and higher expression of anti-apoptotic genes than MDA-MB-231 cells (Table 1). The results showed that 13-EBR treatment induced the expression of Bcl-2 family proteins and the activation of caspase-9, -3, and PARP in both MDA-MB-231 and RT-R MDA-MB-231 cells, which suggests that 13-EBR promotes RT-R TNBC apoptosis through the mitochondria-related intrinsic pathway. However, we did not observe the activation of caspase-8, which suggested that the extrinsic pathway is not involved in 13-EBR-evoked TNBC and RT-R TNBC apoptosis (Figure 4A,B). Although the induction of apoptosis in RT-R MDA-MB-231 cells is complicated due to the lower expression levels of pro-apoptotic genes and the higher expression levels of anti-apoptotic genes than in the parental cells, 13-EBR showed effective anticancer effects in both MDA-MB-231 and RT-R MDA-MB-231 cells.

**Figure 5:**
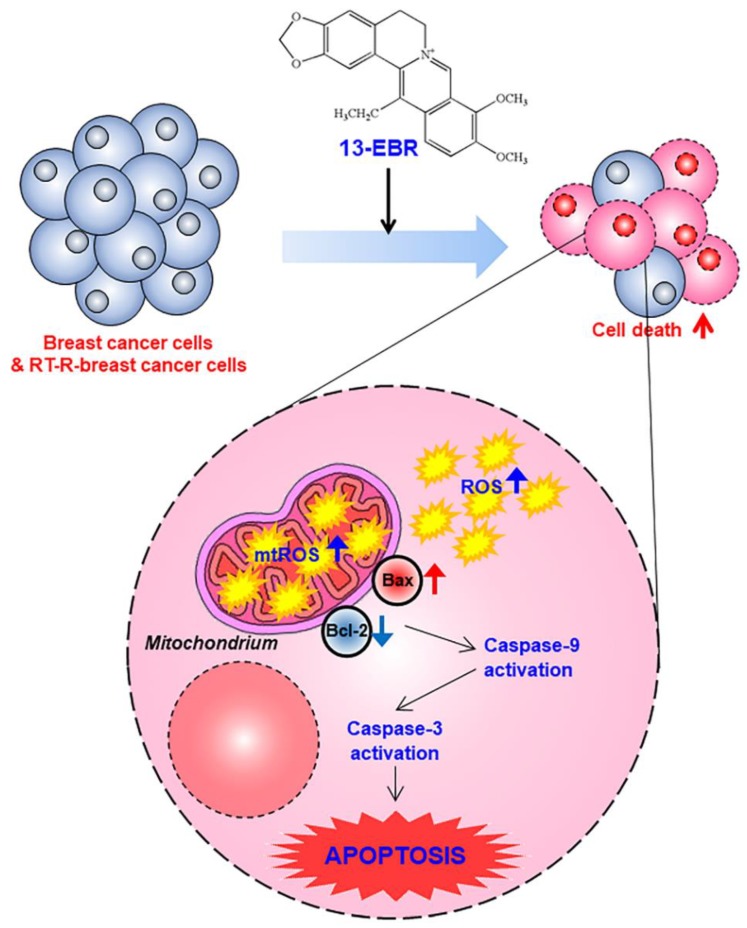
A proposed mechanism via which 13-EBR exhibits anticancer effects in triple-negative breast cancer (TNBC) and RT-R TNBC. Treatment with 13-EBR exhibits anticancer effects through the suppression of cell proliferation and colony-forming ability and by inducing apoptosis through the mitochondria-mediated signaling pathway in RT-R MDA-MB-231 cells, as well as MDA-MB-231 cells.

Taken together, we demonstrated that 13-EBR has antiproliferative and pro-apoptotic effects in both TNBC and RT-R TNBC cells through the induction of intracellular ROS production and the mitochondria-mediated apoptosis pathway (Figure 5). Although the effects of 13-EBR were not specific to RT-R breast cancer cells, it is meaningful that 13-EBR could suppress cell proliferation and colony formation and could induce cellular apoptosis in RT-R MDA-MB-231 cells, which had higher colony-forming abilities and showed lower expression of pro-apoptotic genes and higher expression of anti-apoptotic genes than MDA-MB-231 cells. These findings provide useful insights for the exploration of new potential therapeutic strategies for both radiotherapy-resistant breast cancer treatment and also TNBC treatment.

## 4. Materials and Methods

### 4.1. Materials

The 13-EBR (Figure 1A) was kindly provided by Prof. Dong-Ung Lee (Dongguk University, Gyeongju, Korea). Antibodies against Bax (ab32503), Bcl-2 (ab692), and caspase-8 (ab25901) were purchased from Abcam (Cambridge, UK), and antibodies against cleaved caspase-9 (#52873) and -3 (#9661) were obtained from Cell Signaling Technology (Beverly, MA, USA). The Cell Counting Kit-8 (CCK-8) reagent was obtained from Dongin Biotech (Seoul, Korea). MitoSOX Red was obtained from Thermo Fisher Scientific (Rockford, IL, USA). Hybond-P^+^ polyvinylidene difluoride membranes and enhanced chemiluminescence (ECL) Western blotting detection reagents were purchased from Amersham Biosciences (Little Chalfont, UK) and Bio-Rad (Hercules, CA, USA), respectively. All other reagents, including an anti-β-actin antibody (A2066), propidium iodide (PI), 4′,6-diamidino-2-phenylindole dihydrochloride (DAPI), and 2’,7’-dichlorodihydrofluorescein diacetate (H_2_DCF-DA), were purchased from Sigma-Aldrich (St. Louis, MO, USA).

### 4.2. Establishment of RT-R MDA-MB-231 Cells and Cell Culture

The human breast cancer cell line MDA-MB-231 was obtained from the Korea Cell Line Bank (Seoul, Korea). RT-R MDA-MB-231 cells were established as described by Ko et al. [22]. Briefly, cells were irradiated with 2 Gy using a 6-MV photon beam that was produced by a linear accelerator (Clinac 21EX, Varian Medical Systems, Inc., Palo Alto, CA, USA) until a final dose of 50 Gy was achieved, which is a commonly used clinical regimen for radiotherapy in patients with breast cancer. RT-R MDA-MB-231 cells were cultured in Roswell Park Memorial Institute (RPMI) 1640 medium supplemented with 10% fetal bovine serum (FBS) (HyClone Laboratories, Logan, UT, USA), 100 IU/mL penicillin, and 10 µg/mL streptomycin (HyClone Laboratories), and then incubated at 37 °C in a humidified atmosphere containing 5% CO_2_ and 95% air. RT-R MDA-MB-231 cells were used within five passages.

### 4.3. Gene Expression Array Analysis

Total RNA was extracted from MDA-MB-231 and RT-R MDA-MB-231 cells using TRIzol reagent (Invitrogen, Carlsbad, CA, USA) according to the manufacturer’s protocol. Gene expression profiling (*Bax*, *Bad*, *cytochrome c*, *Bcl-2*, *Bcl-2A1*, and *Mcl-1*) was performed with QuantiSeq 3′ messenger RNA sequencing (mRNA-Seq) Service (Ebiogen, Seoul, Korea). Total proteins were also extracted from MDA-MB-231 and RT-R MDA-MB-231 cells using radioimmunoprecipitation assay (RIPA) buffer (0.1% NP-40, and 0.1% sodium dodecyl sulfate in phosphate-buffered saline (PBS)) containing a protease inhibitor cocktail (Sigma-Aldrich). Protein expression profiling (cleaved caspase-3 and -7) was analyzed by a Signaling Explorer Antibody Array (Ebiogen).

### 4.4. Cell Proliferation Assay

Cell proliferation was analyzed using a CCK-8 assay. The cells were seeded in 96-well flat-bottom plates (Thermo Fisher Scientific) and then treated with the indicated doses of 13-EBR and incubated at 37 °C. Then, 10 µL/well of CCK-8 reagent was added at 24, 48, and 72 h and incubated for 30 min at 37 °C. The optical density of each well was measured at a wavelength of 450 nm using a microplate reader (Tecan, Männedorf, Switzerland).

### 4.5. Colony-Formation Assay

MDA-MB-231 or RT-R MDA-MB-231 cells (1 × 10^3^) were seeded in six-well flat-bottom plates (Thermo Fisher Scientific) and treated with the indicated doses of 13-EBR and then incubated at 37 °C. The culture medium was discarded following 24 h and replaced with fresh complete medium every 2–3 days. After 10 days, the medium was discarded, and each well was carefully washed with PBS. The colonies were fixed for 10 min in absolute methanol and then stained with 0.1% Giemsa staining solution at room temperature, and the number of colonies was counted.

### 4.6. Detection of DNA Fragmentation

Cells were seeded on a cover slip that was mounted onto a self-designed perfusion chamber (SPL, Gyeonggi-do, Korea) and then stimulated with 13-EBR at the indicated doses for 24 h at 37 °C. The cells were fixed with 4% formaldehyde (Sigma-Aldrich) for 5 min at 4 °C and then incubated with 0.1% Triton X-100 (Sigma-Aldrich) for permeabilization at 4 °C. After 10 min, the cells were washed with PBS, stained with DAPI, and the fragmented DNA was detected under a fluorescence microscope.

### 4.7. Flow Cytometric Analysis

For the analysis of the cell-cycle profiles, cells stimulated with 13-EBR for 24 h were fixed with 70% ethanol overnight at −80 °C and then washed with ice-cold PBS. Whole cells were then stained with PI solution (10 mM Tris (pH 8.0), 1 mM NaCl, 0.1% NP40, 0.7 µg/mL RNase A, and 0.05 mg/mL PI) in the dark for 30 min at room temperature and then analyzed using a fluorescence-activated cell sorting (FACS) Calibur™ system (Becton Dickinson Bioscience, San Jose, CA, USA).

### 4.8. Measurement of Intracellular ROS and Mitochondrial ROS

Cells were seeded in 96-well plates and then treated with 50 µM 13-EBR for the indicated times. Following treatments, the cells were incubated with 5 µM H_2_DCF-DA (for 30 min) or 5 µM MitoSOX Red (10 min) to determine the intracellular total ROS or mitochondrial ROS, respectively, in the dark. After incubation, the cells were washed three times with PBS. The fluorescence intensity was measured at an emission wavelength of 485 nm and an excitation wavelength of 535 nm for intracellular ROS, or at an emission wavelength of 510 nm and an excitation wavelength of 580 nm for mitochondrial ROS using a microplate fluorescence reader (Tecan).

### 4.9. Western Blot Analysis

For the isolation of total cell extracts, we washed cells with ice-cold PBS and lysed them in RIPA buffer containing a protease inhibitor cocktail. The suspension was centrifuged at 13,000 rpm for 15 min, and nuclear proteins were obtained by further centrifugation at 13,000 rpm for 5 min. For the isolation of total cell extracts, cells were lysed in RIPA buffer (0.1% NP-40 and 0.1% sodium dodecyl sulfate in PBS) containing a protease inhibitor cocktail. Approximately 50–70 μg aliquots of protein were subjected to 10% sodium dodecyl sulfate polyacrylamide gel electrophoresis and transferred onto Hybond-P^+^ polyvinylidene difluoride membranes. The membranes were incubated with the indicated antibodies, and the bound antibodies were detected with horseradish peroxidase (HRP)-conjugated secondary antibodies and an ECL western blotting detection reagent (Bionote, Gyeonggi-do, Korea).

### 4.10. Statistical Evaluations

The treatment groups were compared using one-way analysis of variance (ANOVA) and a Newman–Keuls post hoc test. A *p*-value < 0.05 was considered statistically significant. All data were evaluated for normality and the homogeneity of variance, and are expressed as the mean ± standard error of the mean (SEM).

## Figures and Tables

**Table 1:** Analysis of gene expression levels between MDA-MB-231 and radiotherapy-resistant (RT-R) MDA-MB-231 cells. Total RNA was extracted from MDA-MB-231 and RT-R MDA-MB-231 cells, and the genes involved in apoptotic cell death were analyzed.

Apoptosis-Related Genes		Fold Change (RT-R MDA-MB-231/MDA-MB-231)
**Pro-apoptotic genes**	*Bax*	0.622
	*Bad*	0.620
	*Cytochrome c*	0.576
	*Cleaved caspase-3 (p17)*	0.363
	*Cleaved caspase-7 (p11)*	0.846
**Anti-apoptotic genes**	*Bcl-2*	0.927
	*Bcl-2A1*	8.036
	*Mcl-1*	1.263

## Supplementary Materials

The following are available online, Figure S1: Effects of BBR and 13-EBR on cell proliferation, colony formation, and apoptosis in breast cancer cells.
