# Supplementary material for: 13-Ethylberberine Induces Apoptosis through the Mitochondria-Related Apoptotic Pathway in Radiotherapy-Resistant Breast Cancer Cells
Source: Molecules. 2019 Jul 4;24(13):2448. doi: 10.3390/molecules24132448 (PMC6651458; doi:10.3390/molecules24132448)
Supplement: Supplementary file 1 [file molecules-24-02448-s001.pdf]

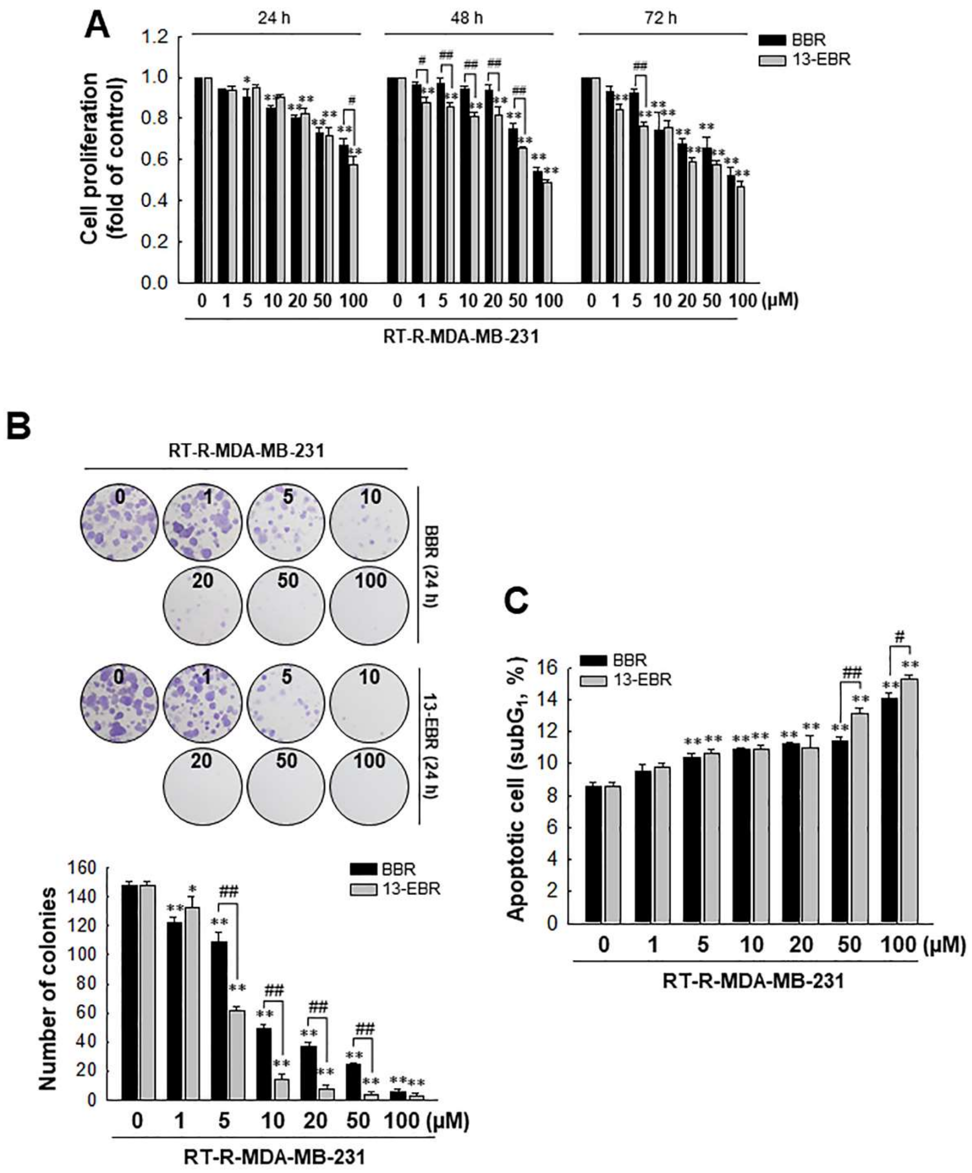


**Supplement.** Effects of BBR and 13-EBR on cell proliferation, colony formation, and apoptosis in breast cancer cells. **A.** RT-R-MDA-MB-231 cells were treated with BBR or 13-EBR at the indicated doses for 24–72 h, then cell proliferations were measured using the CCK-8 reagent. The values represent the mean ± SEM of 3 independent experiments. ** $p < 0.01$, * $p < 0.05$ compared to the control of each treatment group of respective time points; ## $p < 0.01$, # $p < 0.05$. **B.** RT-R-MDA-MB-231 cells (1,000 cells/well) were seeded in 6-well plates, then stimulated with BBR or 13-EBR for 24 h at the indicated doses. Following treatment, colony formation assay was performed. The values represent the means ± SEM of 3 independent experiments. ** $p < 0.01$, * $p < 0.05$ compared to the control of each treatment group; ## $p < 0.01$. **C.** RT-R-MDA-MB-231 cells were treated with BBR or 13-EBR at indicated doses for 24 h, and then apoptotic cells were determined by analyzing the $subG_1$ phase. The values represent the means ± SEM of 3 independent experiments. ** $p < 0.01$ compared to the control of each treatment group; ## $p < 0.01$, # $p < 0.05$.
